# Recurrent and Sustained Viral Infections in Primary Immunodeficiencies

**DOI:** 10.3389/fimmu.2017.00665

**Published:** 2017-06-19

**Authors:** Melanie A. Ruffner, Kathleen E. Sullivan, Sarah E. Henrickson

**Affiliations:** ^1^The Children’s Hospital of Philadelphia, Philadelphia, PA, United States

**Keywords:** virus, primary immunodeficiency, morbidity, herpes, papillomavirus, norovirus, enterovirus

## Abstract

Viral infections are commonplace and often innocuous. Nevertheless, within the population of patients with primary immunodeficiencies (PIDDs), viral infections can be the feature that drives a diagnostic evaluation or can be the most significant morbidity for the patient. This review is focused on the viral complications of PIDDs. It will focus on respiratory viruses, the most common type of viral infection in the general population. Children and adults with an increased frequency or severity of respiratory viral infections are often referred for an immunologic evaluation. The classic teaching is to investigate humoral function in people with recurrent sinopulmonary infections, but this is often interpreted to mean recurrent bacterial infections. Recurrent or very severe viral infections may also be a harbinger of a primary immunodeficiency as well. This review will also cover persistent cutaneous viral infections, systemic infections, central nervous system infections, and gastrointestinal infections. In each case, the specific viral infections may drive a diagnostic evaluation that is specific for that type of virus. This review also discusses the management of these infections, which can become problematic in patients with PIDDs.

## Introduction

Frequent infections are a common reason for physician visits. Distinguishing a pattern or a type of infection that suggests an immunodeficiency as opposed to part of the normal susceptibility to infection can be a challenge. Common causes of recurrent infections are allergies, anatomical contributions, secondary immune deficiency, and an unusual burden of exposures. Primary immunodeficiencies (PIDDs) are much less common and therefore difficult to appreciate during the wealth of infections that are typically seen in a physician’s practice. During the first 5 years of life, children can experience six to eight respiratory tract infections per year. These tend to peak in the winter months and daycare attendance, exposure to smokers, and atopy can increase this frequency significantly (1–4). Respiratory tract infections in adults are somewhat less common; however, three to five respiratory tract infections per year in adults are typical (5). Recurrent sinus infections, pneumonia, and bronchitis are common signs of an immunodeficiency, recognizing that frequent bacterial infections of the respiratory track are often a harbinger of antibody disorders, the most common type of primary immunodeficiency. This review will address recurrent and sustained viral infections for which there are fewer studies to assist the physician in the identification of patients with potential immunodeficiency. This review will address unusual viral respiratory tract infections, systemic viral infections, infections of the brain and meninges, and cutaneous viral infections. Unusual viral infections can be a sign or complication of PIDD. There are several excellent reviews that address the overall approach to suspected PIDD (6–8). Bacterial infections are generally highlighted, and therefore this review will focus on unusual and severe viral infections.

## Respiratory Viral Infections in PIDD

**Figure 1:**
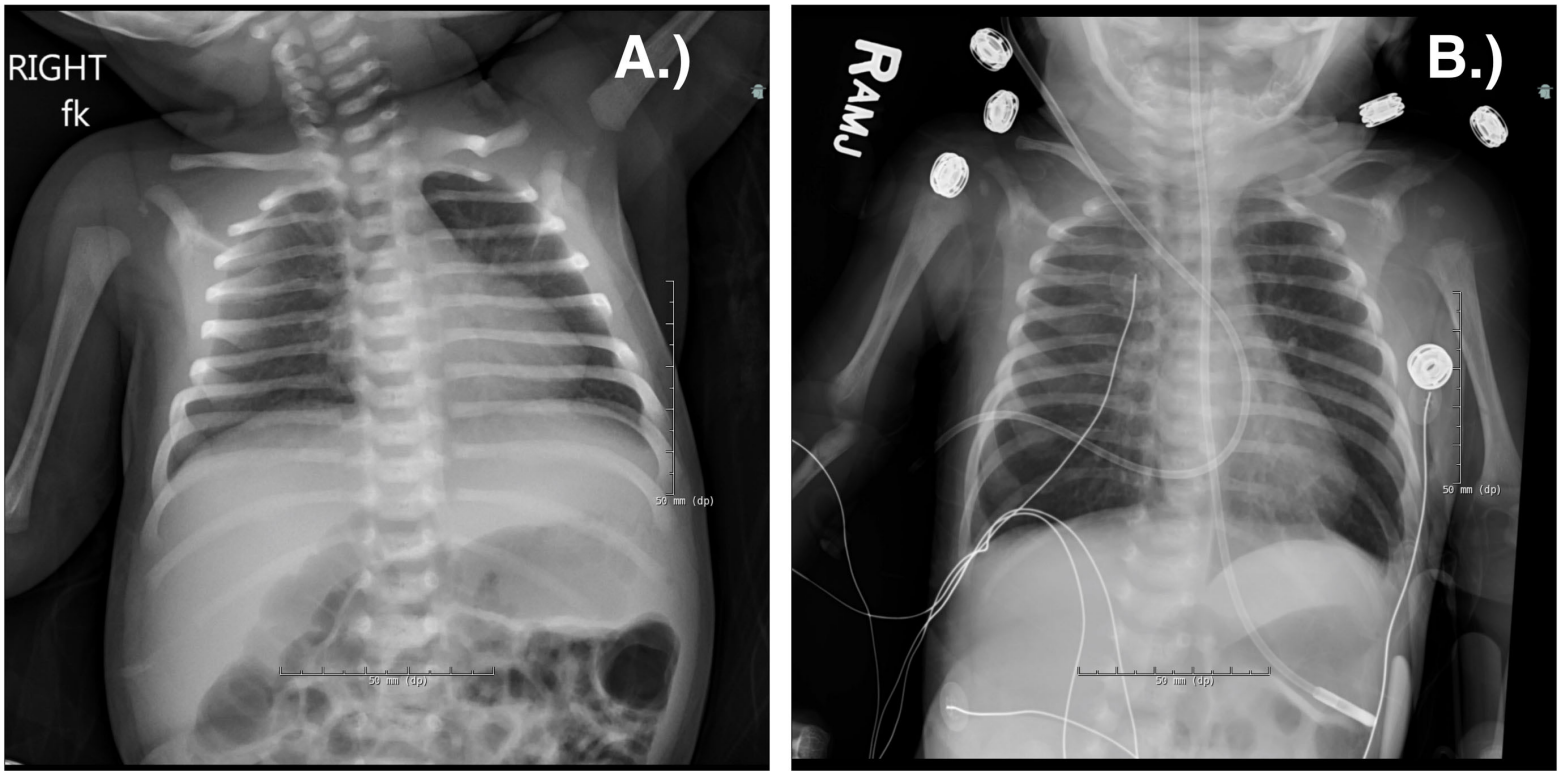
Chest radiograph of a term male infant with X-linked severe combined immune deficiency and RSV pneumonitis, which was rapidly fatal despite adjunctive use of IVIG and inhaled ribavirin. He was treated with an infusion of maternal haplo-identical hematopoietic stem cells at 18 days of life. He was admitted at 10 days old **(A)** and died at 27 days old **(B)** due to worsening respiratory status. Note the absent thymus.

Respiratory viruses are extremely common in most patients with PIDDs (9, 10). In most cases, they represent nuisance infections that can be a predisposing condition leading to bacterial superinfection. In patients with antibody defects, respiratory infections fall into this category. Although defense against recurrence of respiratory tract viruses is mediated largely by antibody, eradication of an infecting virus is mediated largely by the T cell compartment. Respiratory viral infections are therefore more significant in patients with T cell immune deficiencies. Today, many newborns with severe combined immune deficiency (SCID) are detected by newborn screening; however, this is not true in all parts of the world, nor is it true in all states in the USA. A study by the PIDTC found that 21% of their cohort had a respiratory infection prior to transplant with the most common being parainfluenza followed by RSV, rhinovirus, and influenza (11). Although other types of infections were more common as presenting features in this cohort, respiratory infections were among the least likely to resolve prior to transplant. All viral infections are typically prolonged in patients with T cell defects. However, in SCID, there are no T cells and a simple respiratory virus will progress relentlessly unless a hematopoietic stem cell transplant (HSCT) allows the infant to develop a competent immune system (Figure 1) (11). The specific pathogens to which children with T cell defects are susceptible include all of those common in the general population. RSV, influenza, and rhinovirus are typically the most prevalent during the respiratory season (12). Coronavirus and metapneumovirus have been increasingly recognized as causing respiratory infections. Exposures will dictate the pattern of viral infections in patients with T cell defects. The severity of disease is a result of both the degree of T cell compromise and the nature of the infecting virus. Any patient with prolonged viral infections is at risk for bacterial superinfection. It is not, therefore, uncommon to see a mixed picture of viral and bacterial infections. Additionally, severe T cell defects are associated with compromised antibody production, also contributing to a mixed infection picture.

T cell defects are associated with a generally increased predisposition to viral infections. IRF7 deficiency, in contrast, is associated with a selective susceptibility to influenza (13). It is otherwise uncommon to see an isolated susceptibility to a respiratory virus. The single reported patient had a severe primary infection with influenza associated with poor production of type I and type III interferons.

### Diagnostic Approach

There are many specific genetic types of immunodeficiencies associated with T cell deficiency, and the diagnostic considerations are different depending on the age of the patient. Infants with a prolonged or severe respiratory viral infection should be evaluated for SCID, and patients of any age with dysmorphic features or other associated features should be evaluated for chromosome 22q11.2 deletion syndrome (14). Most states now have a newborn screening program to detect SCID. The results can be accessed to identify significant T cell lymphopenia in early infancy. Newborn screening has significantly improved survival of infants with SCID. There are, however, significant T cell disorders not identified by this test, and therefore, T cell disorders still represent a concern in the setting of a prolonged viral infection. T cell enumeration is often the quickest way to screen for T cell defects. The vast majority of T cell deficiency conditions will have low T cell numbers or at least low CD4/CD45RA (naïve) T cell counts. Additional studies include proliferative studies, exclusion of HIV, and sequencing panels to identify inborn errors of immunity.

### Management

Management of respiratory tract infections in non-SCID T cell defects is largely supportive with optimization of bronchodilators, antiviral therapy if available and attention to nutrition (12). Management of respiratory tract infections in SCID is highly problematic. There is a race to replace the immune system before the virus can progress to the point of no return. This race is highly dependent on the type of transplant donor, type of conditioning, and type of transplant, but respiratory infections clearly impact the transplant outcome (11). Any adjunctive measure to improve respiratory status should be sought.

## Systemic Viral Infections in PIDD

Children with severe T cell defects are also susceptible to systemic viral infections. Patients with SCID are extremely susceptible to progressive infection with cytomegalovirus (CMV) as well as other systemic viral infections. Infants with suspected SCID should be protected from exposures such as breast milk, transfusions, potentially infected siblings, live viral vaccines, and caregivers.

There is another circumstance in which susceptibility to gamma-herpes viruses such as CMV and Epstein–Barr virus (EBV) occurs. Table 1 lists conditions in which gamma-herpes virus susceptibility dominates the clinical picture. Patients with cytolytic T cell defects (with or without concomitant NK cell defects) exhibit a unique susceptibility to these gamma-herpes viruses (15). In some circumstances, the susceptibility is almost entirely limited to susceptibility to either severe mononucleosis or hemophagocytic lymphohistiocytosis (HLH). HLH is characterized by excessive immune activation and can be diagnosed either by a molecular diagnosis consistent with HLH or clinically when patients meet five out of eight criteria: fever, splenomegaly, cytopenias affecting two or more blood lineages, hypertriglyceridemia and/or hypofibrinogenemia, hemophagocytosis, low/absent natural killer cell activity, hyperferritinemia, and high soluble interleukin-2 receptor levels (16). Any patient presenting with exceptionally severe mononucleosis or HLH should be screened for the HLH defects.

**Table 1:** EBV susceptibility.

Phenotype	Gene defect	Viral susceptibility	Other features
EBV viremia	*ITK*	EBV	Lymphoma
EBV viremia	*MAGT1*	EBV	Lymphoma
EBV viremia	*CD27*	EBV	Low IgG
EBV viremia	*CORO1A*	Many viruses	Lymphoma
EBV HLH	*SH2D1A*	EBV	Lymphoma, dysgammaglobulinemia, and vasculitis
EBV HLH	*XIAP*	EBV	Hypogammaglobulinemia
EBV lymphoma	*MCM4*	EBV, CMV	Malignancy, short stature, adrenal insufficiency
Primary familial HLH	*PRF1, UNC13D, STX11, STXBP3*	EBV, CMV, others	
Pigmentary dilution with HLH	*LYST, RAB27A, AP3B1, BLOC1S6*	EBV, CMV, others	Pigmentary dilution
EBV susceptibility with broad infectious susceptibility	Leaky SCID, most combined immunodeficiencies, *WASP, WIPF1, PLCG2, PRKCD, ORAI1, STIM1, IKBKG, CASP8, STAT1* GOF, *DOCK8, GATA2*	Many viral susceptibilities	Gene dependent

*GOF, gain of function; LOF, loss of function; EBV, Epstein–Barr virus; HLH, hemophagocytic lymphohistiocytosis; CMV, cytomegalovirus; SCID, severe combined immune deficiency; IgG, immunoglobulin G*.

A second phenotype with susceptibility to EBV has a smoldering or even asymptomatic presentation. These patients generally have an increased risk of lymphoma due to chronic EBV, but the manifestations of EBV may be subtle or absent. The X-linked disorder due to deficiency of *MAGT1* is associated with a mild susceptibility to other infections but chronic EBV. Similar conditions include deficiencies of *CD27, CTPS1, RASGRP1, CD70*, and *MCM4*. The importance in recognizing this group is due to their unpredictable capacity to control EBV and the risk of lymphoreticular malignancies.

Nearly all of the leaky SCID types and the combined immunodeficiencies are associated with an increased risk of CMV and EBV (17, 18). This set of disorders can have a broad phenotype including Omenn’s phenotype, autoimmunity, granulomas, and infections (18–23). In these patients, EBV and CMV can drive progression to malignancy, and they require careful monitoring. Leaky SCID has been defined as T cell lymphopenia (CD3 300–1,500 cells/mm^3^); functional impairment as defined by proliferative responses, absence of maternal engraftment, and most often having identified hypomorphic mutations in genes associated with SCID.

### Diagnostic Approach

When the consideration is prolonged infection with gamma-herpes viruses without HLH, T cell counts and function can be helpful as supporting information but often genetic testing is the quickest approach. For HLH disorders, enumerating the HLH criteria is a useful exercise. CD163 staining of the bone marrow can be a sensitive way to identify active hemophagocytosis, but this is not required for the diagnosis (15, 16, 24). Additional maneuvers are measurement of IL-2R in the serum and CD107a on the surface as a marker for degranulation. HLH can occur without an underlying PIDD, and thus genetic analysis is often central to the management. Nearly always an underlying PIDD will require HSCT as definitive therapy, whereas HLH due to uncommon infections such as *Leishmania*, certain influenza viruses, and arboviruses will not require HSCT.

### Management

Management of systemic viral infections relies on the availability of antiviral compounds. For CMV, therapy is often begun with ganciclovir or valganciclovir (25, 26). Foscarnet may be added if the virus is resistant or progressive in spite of adequate ganciclovir (27). Bone marrow toxicity from ganciclovir may also require a change to foscarnet. EBV in some cases is treated with rituximab to eliminate one important reservoir of virus (28). When HLH is present, a systemic approach to stabilize the patient and treat the underlying inflammation is essential (29). Risks and benefits of antiviral therapy must be carefully weighed as all approaches can have significant adverse events. Management decisions are often impacted by subsequent transplant strategies.

## Chronic Viral Skin Infections in Primary Immunodeficiency

Cutaneous manifestations are common in PIDD. As many as two-thirds of the patients have cutaneous manifestations at some point. Atopy, infection, and inflammatory lesions have all been described, and there may be interplay between the features (30). Awareness of common skin infections is important both to aid in the early diagnosis and also in the treatment of potentially life-threatening infections that can begin in the skin. Bacterial infections are one of the most common findings in PIDD. For example, folliculitis, abscesses, and impetigo are typical in neutrophil defects. Similarly, a significant subset of PIDD diagnoses is associated with fungal infections. These can be seen both in T cell defects as well as defects of the myeloid compartment. Chronic mucocutaneous candidiasis is most often due to defects that affect the Th17 cell production or function. These diseases generally do not overlap those with a susceptibility to cutaneous viral infections. One exception is *STAT1* gain-of-function (GOF) mutations that render patients susceptible to a broad range of cutaneous infections. Viral infections of the skin are not nearly as common but are much more suggestive of PIDD. Severe herpes infections and papillomavirus are particularly characteristic of PIDD and can become the most notable feature in a patient. Chronic herpes virus and papillomavirus, in turn, predispose to cutaneous carcinoma and surveillance becomes important for this evolution. In this section, we will provide a brief synopsis about the individual disorders associated with susceptibility to papillomavirus.

### Papillomaviridae

**Figure 2:**
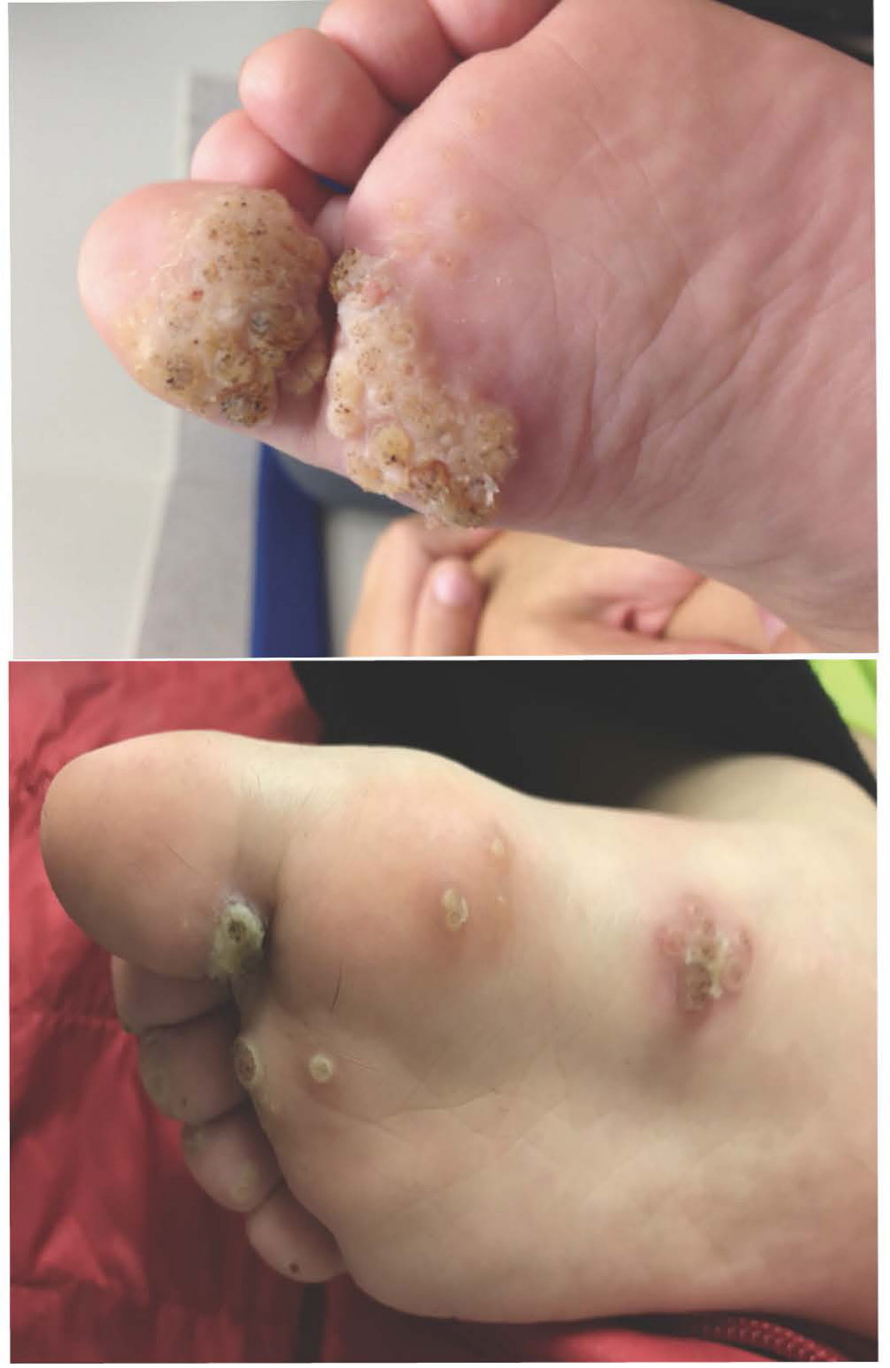
Bilateral plantar warts on a patient who had experienced 5 years of immune suppression for a cardiac transplant. Deep palmoplantar warts such as those in the top panel are referred to as myrmecila and can be painful. The small black markings are characteristic and represent small blood vessels that have grown into the exophytic lesion. Photo credit: Marissa J. Perman, MD.

There are more than 200 strains of human papillomavirus (HPV). The diverse strains have variable malignant potential and tissue tropism. HPV causes warts in the general population with an incidence of cutaneous warts (Figure 2) that range from 1 to 12% (31–33). School age children have been estimated to have a cutaneous warts prevalence of over 40% (34). While sexually active women 20–24 years of age have a prevalence of genital papillomavirus of nearly 50% (35), clinically significant genital warts occur in only 5% of women (36). Genital HPV infection has been associated with malignant transformation, leading to the development of the first vaccine intended to prevent cancer. Worldwide, 5% of cancer is caused by HPV (37). Nearly all T cell disorders can be associated with increased susceptibility to warts; however, there is a small group of PIDDs that have warts as a cardinal feature (38). In these patients, the warts are recurrent, severe, and resistant to therapy. In most cases, the specific papillomaviruses are identical to those in the general population, and the appearance of the warts is the same as in the general population. In epidermodysplasia verruciformis (EV), however, there is a broader susceptibility to HPV types. In addition, the cutaneous manifestations are often atypical. Table 2 compares the features of the different conditions described below.

**Table 2:** Cutaneous viral infections in primary immunodeficiencies.

Viral family	Virus	Increased susceptibility in which PID	Other features
Papillomaviridae	HPV	Ataxia telangiectasia; *DOCK8*; EV (*EVER1, EVER2, RHOH*; *LCK*); *GATA2*; Idiopathic T cell lymphopenia; Netherton syndrome; *STK4/MST1*; WHIM (*CXCR4*); WILD, *CARMIL2/RLTPR*, Clouston’s syndrome	EV: warts are often flat, appearing as actinic keratosis or seborrhea-like lesions and can have increased susceptibility to unusual HPV strains. No other infectious susceptibility DOCK8, GATA2: also include susceptibility to HSV. Progressive lymphopenia seen
Herpesviridae	HHV8/KSHV	IFNGR1, OX40	Susceptibility to mycobacteria
	HSV	*DOCK8*; *GATA2*; *NEMO*; *STAT1 GOF*; *STK4*; *CXCR4*; Wiskott–Aldrich syndrome (WAS)	*DOCK8, NEMO, STAT1* GOF: broad infectious susceptibility *CXCR4*: pancytopenia, abnormal neutrophils WAS: thrombocytopenia, eczema
	VZV	*DOCK8*; *GATA2*; *STAT3* GOF; *IFNGR1*; *RHOH*; *STAT*1 GOF; *STK4*	*DOCK8, NEMO, STAT1* GOF: broad infectious susceptibility *CXCR4*: pancytopenia, abnormal neutrophils WAS: thrombocytopenia, eczema
Poxviridae	MCV	*DOCK8*; *GATA2*; *IKBKG*; *STAT1* GOF; *STK4*; *CXCR4*; *CARMIL2/RLTPR*	*DOCK8, IKBKG, STAT1* GOF: broad infectious susceptibility *CXCR4*: pancytopenia, abnormal neutrophils WAS: thrombocytopenia, eczema
	Orf virus	*STAT1* GOF	Broad infectious susceptibility

*GOF, gain of function; LOF, loss of function; HPV, human papilloma virus; HSV, herpes simplex virus; EV, epidermodysplasia verruciformis; WHIM, warts, hypogammaglobulinemia, infections, and myelokathexis*.

#### *DOCK8* Deficiency

Patients with *DOCK8* deficiency have a complex combined immunodeficiency secondary to disrupted cytoskeletal rearrangement (39). This includes an inability to properly assemble the immune synapse that fosters the signaling cascades required for lymphocyte memory differentiation (40). Lymphocyte migration through tissues is also compromised, contributing to the susceptibility to cutaneous infections (39). T cell counts are typically low for age, and there is impaired memory differentiation that may be progressive with age (41). Clinical features resemble that seen in Wiskott–Aldrich syndrome (WAS) including low IgM and elevated IgE and IgA. Patients have significant atopy, infections, impaired specific antibody responses and poor memory B cell responses, increased rates of malignancy, elevated IgE, and eosinophilia and are highly susceptible to cutaneous viral infections. Early in life, the atopic manifestations may dominate while the infectious susceptibility evolves. Skin infections are most often caused by HPV (including increased risk of malignant transformation of skin lesions), herpes simplex virus (HSV), molluscum contagiosum virus, and varicella zoster virus (42). HSCT has been shown to be curative (43).

#### Epidermodysplasia Verruciformis

The term EV refers to a group of disorders in which patients are susceptible to beta-HPV with severe diffuse warts, and there is a striking increase in the rate of skin carcinomas (44). *EVER1/TMC6* and *EVER2/TMC8* inactivating mutations cause autosomal recessive (AR) EV. EVER proteins are intracellular zinc transporters, and mutations lead to altered cell activation and a more permissive environment for HPV. Interestingly, the only infectious susceptibility is to HPV. Most patients present in childhood, but the appearance of the warts can lead to misdiagnosis as seborrhea or tinea versicolor. The warts are generally worse on sun-exposed skin for reasons that are not clear. The specific HPV types are not necessarily those seen in the general population as wart-associated. The therapy in EV is usually local control since the susceptibility does not relate to hematopoietic cell dysfunction; there is no role for HSCT.

#### *LCK* Deficiency

*LCK* deficiency causes atypical EV with CD4 T cell deficiency as well as recurrent pneumonia and severe warts complicated by non-melanoma skin cancer (45). To date, only a single patient with *LCK* deficiency has been described, leading to uncertainty in the full spectrum of infectious susceptibility. Therapy is not clear for the same reason, but HSCT would be expected to be curative.

#### *CARMIL2/RLTPR* 

Three Norwegian families have been identified with increased viral cutaneous infections (warts and molluscum contagiosum) as well as dermatitis and pneumonia. The four affected family members were found to share a single variant in this gene, with some evidence for a role in T cell activation (46).

#### *RHOH* Deficiency

Mutations in the *RHOH* gene (an atypical Rho GTPase) cause susceptibility to EV-type HPV strains, due to alterations in T cell activation and homing (47). Naïve T cell counts are low, and there is poor skin homing of T cells, with an increase in effector memory T cells in the setting of altered T cell receptor signaling. *RHOH* deficiency infectious susceptibility was largely limited to HPV in the two siblings identified. In mice, the defect was correctable by transfer of wild-type bone marrow, suggesting that this is a potential treatment.

#### *IKBKG* Deficiency (*NEMO*)

Hypomorphic mutations in the central kinase of the canonical NFκB pathway lead to increased susceptibility to warts (48). This PIDD is associated with fine sparse hair, dental defects, and a broad susceptibility to infections including opportunistic infections. The spectrum of phenotypes is among the broadest among PIDDs. Lymphedema, osteopetrosis, susceptibility to pneumocystis and other fungi, mycobacterial susceptibility, and viral susceptibility call all be associated with *IKBKG* deficiency. Inflammatory bowel disease is also common. Cutaneous viral infections are common in this population but are not usually the dominant feature. Among the PIDD in this section, this is a disorder where the benefit of HSCT is unclear because HSCT outcomes have been poor.

#### *GATA2* Deficiency

*GATA2* mutations yield a complex set of phenotypes. Patients tend to have monocytopenia and low NK and B cell counts and have susceptibility to HPV, among other infections (49). The laboratory defects are progressive with age and correlate with increasing infection burden. Clinical features include lymphedema, risk of malignancy, and pulmonary alveolar proteinosis. Infections include fungal infections and cutaneous viral infections. The cutaneous viral infections can be the most prominent feature but there is a wide-ranging phenotypic heterogeneity (50, 51). The cutaneous viral infections and genital HPV are associated with a high rate of malignant transformation.

#### *STAT1* GOF

Patients with *STAT1* GOF mutations can present with dramatic autoimmunity, usually including enteropathy and endocrinopathies, or they can have a picture with a pronounced infectious susceptibility. Susceptibility to candida is common, but cutaneous viral infections can also be problematic (52, 53). The viral infections can become more problematic as immune suppression is used to control the enteropathy.

#### Netherton Syndrome

Patients with Netherton syndrome (secondary to *SPINK5* mutations) demonstrate congenital ichthyosis and have higher susceptibility to EV-associated HPV strains (53, 54). Although Netherton syndrome can have a mild humoral immune deficiency, the susceptibility to warts seems to be a result of disrupted local response to infection (55). The warts are generally controllable with local measures.

#### *MST1* Deficiency

Serine–threonine kinase 4 (STK4), encoded by a gene called *MST1*, affects the FOXO1 transcription factor and thus impacts T cell lifespan and has been shown to increase susceptibility to HPV infections (56), as well as causing B cell lymphopenia. The phenotype also includes susceptibility to EBV and cutaneous viral infections.

#### Warts, Hypogammaglobulinemia, Infections, and Myelokathexis (WHIM) Syndrome

Warts, hypogammaglobulinemia, infections, and myelokathexis syndrome is caused by an autosomal dominant (AD) GOF mutation in *CXCR4*, which leads to increased susceptibility to HPV (57, 58). Varying degrees of pancytopenia can be seen, and hypogammaglobulinemia occurs in many (but not all) patients. Warts can be the most prominent susceptibility, but neutropenia and hypogammaglobulinemia can drive diverse infectious susceptibilities. COPD and cutaneous carcinoma have been observed (59). WHIM can be treated with plerixafor or topical control measures for the warts.

#### WILD Syndrome

WILD (warts, depressed cell-mediated immunity, primary lymphedema, and anogenital dysplasia) is also correlated with severe warts without a known genetic etiology (60). Of note, this diagnosis does not lead to EV-defining HPV strain infections. A recent study demonstrates a case of a patient whose warts improved after quadrivalent HPV vaccination (61).

#### Clouston Syndrome

An ectodermal dysplasia syndrome with alterations in hair and nails (not generally in teeth) which can present with eccrine syringofibroadenomatosis that is reminiscent of EV and is associated with HPV infection (62).

#### Other Settings

Idiopathic CD4 T cell lymphopenia is associated with increased risk of cutaneous warts (63), and SCID patients post-HSCT can have an increased risk of warts, especially with specific underlying mutations (64, 65).

#### Management

Management of cutaneous warts typically progresses from low level removal approaches involving topical therapy (freezing, electrosurgery, curettage, laser, chemical softening, and cantharidin) to immune stimulants (imiquimod and antigens), bleomycin, and antiviral therapy including cidofovir (66). Genital warts are treated with conceptually the same approach. Management of genital warts also includes special considerations for pregnancy, partners, and screening for malignancy (67–69). Interferon a2 and GM-CSF have been used successfully for papillomavirus (70, 71). In severe cases, therapy can be very unsatisfying. HSCT can result in prompt eradication, though *JAK3* mutations and common γ chain SCID can have significant warts post-HSCT, which may be in part due to poor NK cell function (64). Topical cidofovir has recently been shown in a case report to be effective in some of these patients (72). In one case, regression was observed after papillomavirus vaccination (61).

### Herpesviridae

The Herpes family of DNA viruses includes nine viruses pathogenic for humans; CMV, EBV, HHV6a/b, HHV7, HSV1, HSV2, and KSHV, not all of which have skin tropism. Almost any type of T cell dysfunction is associated with an increased frequency of cutaneous HSV. The PIDD with a high likelihood of chronic or disfiguring cutaneous herpes are *DOCK8, GATA2, NEMO/IKBKG, STK4, WAS, STAT1* GOF, and WHIM (*CXCR4* mutations) as described above in association with warts. These same syndromes can manifest with severe primary varicella and an increased frequency and severity of zoster (73). Varicella and zoster can also be seen in settings with altered proportions of T cell subsets, as in *STAT3* LOF deficient patients (AD-hyper IgE syndrome) (74) or cytokine signaling defects as seen in *IFNGR1* mutations (75). Thus, susceptibility to herpes viruses occurs in a broad range of T cells defects and is often part of a complex of susceptibility to many cutaneous viral infections. Herpes viruses can be particularly persistent and cause significant morbidity and mortality. Carcinoma is a feared consequence of recurrent or persistent infection and can be difficult to distinguish from persistently infected skin.

#### Management

Management includes acyclovir, valacyclovir, or famciclovir as initial options. IL-2 therapy has been used successfully in WAS (76). In WAS, it specifically improves NK cell function (77). Type I and type II interferons have been used successfully in model systems (78, 79). Small studies of non-PIDD populations have supported its use in patients (80–83). Management should focus on prevention of recurrences and healing of cutaneous lesions. Malignant transformation relates in complex ways to persistence of infection.

### Poxviridae

Poxviridae are double stranded DNA viruses. Molluscipoxvirus (i.e., molluscum contagiosum virus or MCV), orthopoxvirus (i.e., smallpox), parapoxvirus, and yatapoxvirus are the four genera that can infect humans. Susceptibility to poxviridae tends to be associated with susceptibility to other cutaneous viral infections. Therefore, many of the disorders described above have an increased susceptibility to poxviridae. Molluscum contagiosum in the most common pox virus infection in humans and in the general population is a self-limited infection with minimal residua (84). Severe molluscum contagiosum can be seen in *DOCK8* deficiency, *STK4* deficiency (56), *GATA2, NEMO, STAT1* GOF, and WHIM syndrome. Molluscum can be a significant issue for patients with WAS, as described in the Herpesviridae section. In addition, Orf virus, found in pasture animals, was found in a single patient with *STAT1* GOF (85). Similar to herpes virus susceptibility described above, molluscum can be seen with nearly any T cell defect and typically arises in that setting as part of a susceptibility to many cutaneous viruses.

#### Management

Initial management of molluscum in a PIDD patient is control of spread through curettage, topical therapy such as salicylate, cantharidin, or immune stimulation with imiquimod (86, 87). Other topical approaches have also been used successfully. Retreatment 2–4 weeks later is often required. If addressed early, spread may be controlled and the outbreak contained. For diffuse disease or disease that spreads despite all attempts at control, type I interferon (interferon a2) has been suggested (88, 89). Intralesional immunotherapy with live antigen has been promoted but is contraindicated in PIDD patients with T cell defects (90, 91).

### Persistent Vaccine-Stain Rubella Infection

Three studies have identified persistent vaccine-strain rubella in patients with moderate T cell defects (91–93). Most of the patients have had ataxia telangiectasia but a wide range of PIDD diagnoses have been seen. Generally, the patients have had sufficient T cell function to be leading relatively normal lives and the immune deficiency might not even be recognized at the time of the MMR vaccine administration. The manifestations have been largely cutaneous granulomas although chronic inflammation at other sites has been observed (91). Persistence of virus due to compromised T cell control and acquisition of mutations that may further impact clearance is the proposed mechanism.

### Diagnostic Considerations for Patients with Cutaneous Viral Infections

The above conditions are derived from defects in T cell, NK cell, and local tissue immunity. It is therefore nearly impossible to systematically screen for gene defects related to cutaneous viral infection susceptibility. A reasonable start is to define T cells both quantitatively and functionally. If that is unrevealing and the phenotype suggests a PIDD, then whole exome sequencing may be appropriate.

## Gastrointestinal (GI) Viral Infections

Chronic diarrhea (>6 weeks) is a frequent finding in PIDD patients. Given that the etiologies of chronic diarrhea in immunodeficient patients can be diverse, it is important to first distinguish if the diarrhea is infectious, malabsorptive, or inflammatory in nature as there are multiple types of PIDD that can present with autoimmune enteropathy or inflammatory bowel disease (94–96). PIDD patients are susceptible to multiple types of GI pathogens, and this section will focus on GI viruses. The concerted action of both the innate and adaptive immune system is necessary for viral clearance (97, 98). Therefore, there are a number of combined immunodeficiency phenotypes that result in susceptibility to GI viral pathogens, which manifest as prolonged illness as well as prolonged asymptomatic viral shedding.

### Norovirus

Persistent infection with norovirus resulting in prolonged viral shedding and symptomatic disease has been noted in patients with SCID and various secondary immunodeficiency states (99). In a series of pediatric PIDD patients, it was the most frequently isolated virus at 20.6%, with patients with SCID, major histocompatibility complex II deficiency, CD40L deficiency, and agammaglobulinemia represented in this series (100). Norovirus shedding can be prolonged in the stool of patients who were immunosuppressed following infection and norovirus can be part of multiple infections in the GI tract (101). In CVID, norovirus infection has been linked to development of severe enteropathy with prolonged viral carriage over the course of years (102). In several patients in this series, clearance of norovirus resulted in normalization of the GI enteropathy.

A concern about norovirus is the great difficulty in public health containment. A patient with PIDD who is shedding for a prolonged period of time is not only themselves at risk but also places those around at risk. Norovirus is a common pathogen in the general population, and exposures are therefore common. Norovirus is spread from the moment of illness to several days after clinical recovery. Both vomit and feces can spread virus. The virus lives on surfaces for up to 20 days, and alcohol-based cleaners are not completely effective. Patients with chronic norovirus should use bleach to clean surfaces. Vigorous hand washing with soap and water is also effective.

### Hepatitis C

In the early 1990s, the United States FDA recommended that hepatitis C positive donors be excluded from the plasma donor pool resulting in loss of neutralizing hepatitis C viral antibodies from IVIG (103). Hepatitis C virus (HCV) infection was subsequently reported from several countries, which was due to the presence of contaminating HCV virus from the small numbers of seronegative HCV positive donors (103–106). The severity of hepatitis seen in immunodeficient patients was variable, but subsets of patients with primary hypogammaglobulinemia were observed to have a more severe course of hepatitis, which in some cases was rapidly fatal (103, 105, 106). Younger age and early treatment with IFN were associated with better overall outcomes (103, 106). Following adoption of PCR screening for HCV and viral inactivation processes with solvent–detergent or pasteurization there have been no subsequent reports of IVIG-associated HCV since 1996 (107). This cautionary tale supports surveillance of PIDD patients who have risk factors for HCV: blood product exposure, including IV drug use, infants born to HCV positive mothers, high-risk sexual behavior, shared personal items with potential blood exposure among HCV positive individuals. Today, therapy for HCV should improve outcomes compared to the cohort in the early 1990s.

### Other GI Viruses

Adenovirus, enterovirus, and rotavirus have been isolated from single PIDD patients with chronic diarrhea, and the true incidence is not known (100). Chronic rotavirus infection has been described in patients with SCID and agammaglobulinemia (108). In patients with immunodeficiency, rotavirus can be poorly contained within the GI tract. On investigation at autopsy, active rotavirus replication has been identified in the liver and kidney of patients with SCID, complete DiGeorge syndrome, and acquired-immunodeficiency syndrome, illustrating poor control of viral replication in the setting of profound immunodeficiency (109). An important consideration is that SCID has been associated with susceptibility to the live rotavirus vaccine (110). Indeed, vaccine-strain illness is cleared only after immune reconstitution (110, 111).

CVID and agammaglobulinemia can rarely have prolonged asymptomatic shedding of vaccine-strain polio following immunization with live-attenuated oral polio vaccine, which can pose risk to other immunocompromised members of the community (112–114). Additionally, central nervous system (CNS) infection can occur in agammaglobulinemia (see below). These are the main reasons that live polio vaccination is no longer used in the USA.

### Management

Care for chronic GI viral infections in PIDD is primarily supportive: optimizing hydration and nutrition. Orally administered immunoglobulin G (IgG) has been demonstrated to be effective as a therapy for chronic infectious diarrhea in antibody deficient patients (115, 116). Oral IgG survives passage through the stomach and is bioavailable (116). Antiviral therapies are untested; however, they could be considered in severe disease.

## Viral Infections of the CNS in PIDD

Viral infections of the CNS confer significant morbidity and mortality in the general population (117–119). Therefore, they are not often considered to be indicators of primary immunodeficiency. There are two circumstances where an infection of the CNS is often associated with a primary immunodeficiency: atypical herpes simplex encephalitis and CNS enteroviral disease.

### Herpes Simplex Encephalitis

Herpes simplex encephalitis in the general population is most typically seen in newborns and is typically caused by herpes simplex type 2. Infection occurs at the time of delivery and infants present in the second week of life with agitation, obtundation, or seizures. Adults can develop herpes simplex encephalitis (120). Underlying immune compromise can be a risk factor for adult-onset herpes simplex encephalitis, and today HIV is the most common associated condition in adults. Herpes simplex encephalitis outside of the neonatal period may therefore suggest an immunodeficiency. Among PIDD, defects in the toll-like receptor pathway are most strongly associated with this infection (Table 3) (121). Approximately 5% of children with herpes simplex encephalitis have defects in the toll-like receptor pathway (122). Patients with these defects may present in childhood or adulthood, and some patients with just keratitis have been described (123). Recognition is important because therapy can be tailored if the defect is known. Surveillance and prevention of relapses is important. Several of these defects are inherited in an AD fashion, and therefore recognition of these PIDD is critical not only for management of the patient but also surveillance for other family members. A population study suggested that there may be additional defects inherited in an AR fashion yet to be defined (123). A key consideration is that the described toll-like receptor pathway defects are due to loss of local control in the CNS. Antibody and T cell responses are normal, and indeed, local mucosal recurrences are uncommon. As a consequence, testing of the hematopoietic cells is not revealing typically.

**Table 3:** Causes of increased susceptibility to herpes simplex encephalitis.

*TLR3* [autosomal dominant (AD)]
*TRIF* [autosomal recessive (AR)]
*UNC93B1* (AR)
*TRAF3* (AD)
*TBK1* (AD)
*IRF3* (AD)
*STAT1* (AR)
*IKBKG* (XL)

### Enteroviral Meningoencephalitis

Viral meningoencephalitis due to a prolonged infection with enterovirus is strongly suggestive of a specific class of PIDD. Enteroviruses are the most common cause of viral meningitis in the general population manifesting as acute onset headache with gradual resolution over days to a few weeks. In patients with agammaglobulinemia, manifestations are quite different (124). These children typically present with regression of developmental milestones. Ataxia or clumsiness may be noted by parents or on examination. Features early on are subtle, and the slow progression can lead to efforts at mitigation with physical therapy or behavioral strategies. In a patient with a known humoral immune deficiency, the index of suspicion should be high and a workup should not be delayed if there are clear neurologic signs or symptoms. CNS infection in patients with agammaglobulinemia has a very poor prognosis. There can be other phenotypes associated with enteroviral disease in patients with agammaglobulinemia; however, CNS infection is the most common. Dermatomyositis and hepatitis have been described and have progressed in some cases to CNS infection. Treatment for enteroviral disease includes high dose immunoglobulin and when available, drugs directed at enterovirus.

A unique subset of CNS enteroviral infections occurs in either SCID or agammaglobulinemia with live-attenuated polio vaccine. Wild-type polio, occurring in three serotypes, has been nearly eradicated. Even early on, it was recognized that the live-attenuated vaccine could cause disease (125) and that patients with hypogammaglobulinemia could excrete virus for years (126, 127). Currently, circulating wild-type polio is seen only in Afghanistan and Pakistan although virus can be isolated in sewage from other countries supporting ongoing risk for immunodeficient individuals (128). Vaccine-associated poliomyelitis can be due to infection of an immune deficient individual and spread to the CNS or to revertants of vaccine-strain virus (129, 130). In the latter case, even normal hosts can have overt paralytic disease. Vaccine-associated poliomyelitis can appear as acute flaccid paralysis or with a meningoencephalitis in immunodeficient individuals. The prognosis has generally been poor (131).

### Diagnostic Approaches

Testing for defects related to herpes simplex encephalitis often involves genetic sequencing although functional analyses are available on a research basis. Table 3 lists the currently recognized genetic causes of susceptibility to herpes simplex encephalitis.

The diagnosis of enteroviral meningoencephalitis in PIDD patients requires a specific description. In a patient with agammaglobulinemia detection of enterovirus is surprisingly difficult. PCR analysis of cerebrospinal fluid or stool (less specific) should be performed. However, it is not unusual for children with agammaglobulinemia and suggestive clinical features to require a brain biopsy for diagnosis. The biopsy tissue can be tested for enterovirus by PCR. In a patient who presents with CNS enteroviral disease, identification of an immune deficiency is critical because of the prognostic implications. The strong association of CNS enteroviral disease with agammaglobulinemia supports a strategy that begins with enumeration of peripheral blood B cells by flow cytometry. Only if that is negative and there are no other secondary immune deficiencies should alternatives such as CD40L or CVID be sought. A reasonable secondary screen would be to measure immunoglobulin levels and responses to vaccines.

### Management

Management of herpes simplex encephalitis requires specific antiviral approaches as well as attention to seizures, increased intracranial pressure, and a comprehensive intensive care approach. Acyclovir delivered intravenously is the cornerstone of management. One should consider a prolonged course of oral therapy after initial management that could include oral acyclovir or valacyclovir because the relapse rate is high in these toll-like receptor pathway defects. The role of steroids is controversial. One small study of immune competent children supported the use of beta-interferon (132), and it could be argued that interferons specifically mitigate the underlying defect in the toll-like receptor pathway disorders.

Management of CNS enteroviral disease in agammaglobulinemia has recently been reviewed (124). In the USA, antiviral drugs are not available, but pocapavir is under study and may become available. High dose IVIG has been proposed as therapeutic, but survival rates remain dismal and functional outcomes are poor.

## Summary

Viral infections are a common cause of morbidity in patients with PIDDs. They can be a clue to the diagnosis when persistent or unusually severe and can represent a significant management challenge.

## Author Contributions

MR, SH, and KS collectively conceived and wrote the manuscript.

## Conflict of Interest Statement

The authors declare that the research was conducted in the absence of any commercial or financial relationships that could be construed as a potential conflict of interest.

## References

[1] Campbell H Acute respiratory infection: a global challenge. Arch Dis Child (1995) 73(4):281–3. 10.1136/adc.73.4.281 7492188 PMC1511346

[2] Gruber C Keil T Kulig M Roll S Wahn U Wahn V History of respiratory infections in the first 12 year among children from a birth cohort. Pediatr Allergy Immunol (2008) 19(6):505–12. 10.1111/j.1399-3038.2007.00688.x 18167154

[3] Monto AS. Viral respiratory infections in the community: epidemiology, agents, and interventions. Am J Med (1995) 99(6B):24S–7S. 10.1016/S0002-9343(99)80307-6 8585553 PMC7172478

[4] Aligne CA Stoddard JJ. Tobacco and children. An economic evaluation of the medical effects of parental smoking. Arch Pediatr Adolesc Med (1997) 151(7):648–53. 10.1001/archpedi.1997.02170440010002 9232036

[5] Kirkpatrick GL. The common cold. Prim Care (1996) 23(4):657–75. 10.1016/S0095-4543(05)70355-9 8890137 PMC7125839

[6] Aghamohammadi A Abolhassani H Mohammadinejad P Rezaei N. The approach to children with recurrent infections. Iran J Allergy Asthma Immunol (2012) 11(2):89–109.011.02/ijaai.89109 22761184

[7] Costa-Carvalho BT Grumach AS Franco JL Espinosa-Rosales FJ Leiva LE King A Attending to warning signs of primary immunodeficiency diseases across the range of clinical practice. J Clin Immunol (2014) 34(1):10–22. 10.1007/s10875-013-9954-6 24241582 PMC3930833

[8] Bousfiha A Jeddane L Al-Herz W Ailal F Casanova JL Chatila T The 2015 IUIS phenotypic classification for primary immunodeficiencies. J Clin Immunol (2015) 35(8):727–38. 10.1007/s10875-015-0198-5 26445875 PMC4659854

[9] Lanari M Vandini S Capretti MG Lazzarotto T Faldella G. Respiratory syncytial virus infections in infants affected by primary immunodeficiency. J Immunol Res (2014) 2014:850831. 10.1155/2014/850831 25089282 PMC4095650

[10] Rezaei N Hedayat M Aghamohammadi A Nichols KE. Primary immunodeficiency diseases associated with increased susceptibility to viral infections and malignancies. J Allergy Clin Immunol (2011) 127(6):1329–41.e2; quiz 42–3. 10.1016/j.jaci.2011.02.047 21514636

[11] Pai SY Logan BR Griffith LM Buckley RH Parrott RE Dvorak CC Transplantation outcomes for severe combined immunodeficiency, 2000–2009. N Engl J Med (2014) 371(5):434–46. 10.1056/NEJMoa1401177 25075835 PMC4183064

[12] Tregoning JS Schwarze J Respiratory viral infections in infants: causes, clinical symptoms, virology, and immunology. Clin Microbiol Rev (2010) 23(1):74–98. 10.1128/CMR.00032-09 20065326 PMC2806659

[13] Ciancanelli MJ Huang SX Luthra P Garner H Itan Y Volpi S Infectious disease. Life-threatening influenza and impaired interferon amplification in human IRF7 deficiency. Science (2015) 348(6233):448–53. 10.1126/science.aaa1578 25814066 PMC4431581

[14] McDonald-McGinn DM Sullivan KE Marino B Philip N Swillen A Vorstman JA 22q11.2 deletion syndrome. Nat Rev Dis Primers (2015) 1:15071. 10.1038/nrdp.2015.71 27189754 PMC4900471

[15] Chandrakasan S Filipovich AH Hemophagocytic lymphohistiocytosis: advances in pathophysiology, diagnosis, and treatment. J Pediatr (2013) 163(5):1253–9. 10.1016/j.jpeds.2013.06.053 23953723

[16] Henter JI Horne A Arico M Egeler RM Filipovich AH Imashuku S HLH-2004: diagnostic and therapeutic guidelines for hemophagocytic lymphohistiocytosis. Pediatr Blood Cancer (2007) 48(2):124–31. 10.1002/pbc.21039 16937360

[17] Speckmann C Doerken S Aiuti A Albert MH Al-Herz W Allende LM A prospective study on the natural history of patients with profound combined immunodeficiency: an interim analysis. J Allergy Clin Immunol (2017) 139(4):1302–10.e4. 10.1016/j.jaci.2016.07.040 27658761 PMC6311415

[18] Filipovich AH Mathur A Kamat D Kersey JH Shapiro RS. Lymphoproliferative disorders and other tumors complicating immunodeficiencies. Immunodeficiency (1994) 5(2):91–112. 8032367

[19] Wahn V Yokota S Meyer KL Janssen JW Hansen-Hagge TE Knobloch C Expansion of a maternally derived monoclonal T cell population with CD3+/CD8+/T cell receptor-gamma/delta+ phenotype in a child with severe combined immunodeficiency. J Immunol (1991) 147(9):2934–41. 1655900

[20] de Villartay JP Lim A Al-Mousa H Dupont S Dechanet-Merville J Coumau-Gatbois E A novel immunodeficiency associated with hypomorphic RAG1 mutations and CMV infection. J Clin Invest (2005) 115(11):3291–9. 10.1172/JCI25178 16276422 PMC1265866

[21] Ehl S Schwarz K Enders A Duffner U Pannicke U Kuhr J A variant of SCID with specific immune responses and predominance of gamma delta T cells. J Clin Invest (2005) 115(11):3140–8. 10.1172/JCI25221 16211094 PMC1242191

[22] Schuetz C Huck K Gudowius S Megahed M Feyen O Hubner B An immunodeficiency disease with RAG mutations and granulomas. N Engl J Med (2008) 358(19):2030–8. 10.1056/NEJMoa073966 18463379

[23] Walter JE Rosen LB Csomos K Rosenberg JM Mathew D Keszei M Broad-spectrum antibodies against self-antigens and cytokines in RAG deficiency. J Clin Invest (2015) 125(11):4135–48. 10.1172/JCI80477 26457731 PMC4639965

[24] Weitzman S. Approach to hemophagocytic syndromes. Hematology Am Soc Hematol Educ Program (2011) 2011:178–83. 10.1182/asheducation-2011.1.178 22160031

[25] Len O Gavalda J Aguado JM Borrell N Cervera C Cisneros JM Valganciclovir as treatment for cytomegalovirus disease in solid organ transplant recipients. Clin Infect Dis (2008) 46(1):20–7. 10.1086/523590 18171208

[26] Boeckh M. Complications, diagnosis, management, and prevention of CMV infections: current and future. Hematology Am Soc Hematol Educ Program (2011) 2011:305–9. 10.1182/asheducation-2011.1.305 22160050

[27] Razonable RR. Antiviral drugs for viruses other than human immunodeficiency virus. Mayo Clin Proc (2011) 86(10):1009–26. 10.4065/mcp.2011.0309 21964179 PMC3184032

[28] Chellapandian D Das R Zelley K Wiener SJ Zhao H Teachey DT Treatment of Epstein-Barr virus-induced haemophagocytic lymphohistiocytosis with rituximab-containing chemo-immunotherapeutic regimens. Br J Haematol (2013) 162(3):376–82. 10.1111/bjh.12386 23692048 PMC3776423

[29] Jordan MB Allen CE Weitzman S Filipovich AH McClain KL. How I treat hemophagocytic lymphohistiocytosis. Blood (2011) 118(15):4041–52. 10.1182/blood-2011-03-278127 21828139 PMC3204727

[30] Langan SM Abuabara K Henrickson SE Hoffstad O Margolis DJ Increased risk of cutaneous and systemic infections in atopic dermatitis – a cohort study. J Invest Dermatol (2017) 137:1375–7. 10.1016/j.jid.2017.01.030 28202403 PMC5660507

[31] Beliaeva TL [The population incidence of warts]. Vestn Dermatol Venerol (1990) 2:55–8. 2343670

[32] Johnson MT Roberts J Skin conditions and related need for medical care among persons 1–74 years. United States, 1971–1974. Vital Health Stat 11 (1978) 212:i–v, 1–72. 741665

[33] Loo SK Tang WY. Warts (non-genital). BMJ Clin Evid (2009) 2009:1710. 21726478 PMC2907820

[34] de Koning MNC Quint KD Bruggink SC Gussekloo J Bouwes Bavinck JN Feltkamp MCW High prevalence of cutaneous warts in elementary school children and the ubiquitous presence of wart-associated human papillomavirus on clinically normal skin. Br J Derm (2015) 172:196–201. 10.1111/bjd.13216 24976535

[35] Hariri S Unger ER Sternberg M Dunne EF Swan D Patel S Prevalence of genital human papillomavirus among females in the United States, the national health and nutrition examination survey, 2003–2006. J Infect Dis (2011) 204(4):566–73. 10.1093/infdis/jir341 21791659

[36] Flagg EW Schwartz R Weinstock H. Prevalence of anogenital warts among participants in private health plans in the United States, 2003–2010: potential impact of human papillomavirus vaccination. Am J Public Health (2013) 103(8):1428–35. 10.2105/AJPH.2012.301182 23763409 PMC4007878

[37] Tota JE Chevarie-Davis M Richardson LA Devries M Franco EL. Epidemiology and burden of HPV infection and related diseases: implications for prevention strategies. Prev Med (2011) 53(Suppl 1):S12–21. 10.1016/j.ypmed.2011.08.017 21962466

[38] Dropulic LK Cohen JI. Severe viral infections and primary immunodeficiencies. Clin Infect Dis (2011) 53(9):897–909. 10.1093/cid/cir610 21960712 PMC3189169

[39] Zhang Q Dove CG Hor JL Murdock HM Strauss-Albee DM Garcia JA DOCK8 regulates lymphocyte shape integrity for skin antiviral immunity. J Exp Med (2014) 211(13):2549–66. 10.1084/jem.20141307 25422492 PMC4267229

[40] Randall KL Lambe T Johnson AL Treanor B Kucharska E Domaschenz H Dock8 mutations cripple B cell immunological synapses, germinal centers and long-lived antibody production. Nat Immunol (2009) 10(12):1283–91. 10.1038/ni.1820 19898472 PMC3437189

[41] Lambe T Crawford G Johnson AL Crockford TL Bouriez-Jones T Smyth AM DOCK8 is essential for T-cell survival and the maintenance of CD8+ T-cell memory. Eur J Immunol (2011) 41(12):3423–35. 10.1002/eji.201141759 21969276 PMC3517112

[42] Zhang Q Davis JC Lamborn IT Freeman AF Jing H Favreau AJ Combined immunodeficiency associated with DOCK8 mutations. N Engl J Med (2009) 361(21):2046–55. 10.1056/NEJMoa0905506 19776401 PMC2965730

[43] Al-Herz W Chu JI van der Spek J Raghupathy R Massaad MJ Keles S Hematopoietic stem cell transplantation outcomes for 11 patients with dedicator of cytokinesis 8 deficiency. J Allergy Clin Immunol (2016) 138(3):852–9.e3. 10.1016/j.jaci.2016.02.022 27130861 PMC5354354

[44] Orth G. Genetics of epidermodysplasia verruciformis: insights into host defense against papillomaviruses. Semin Immunol (2006) 18(6):362–74. 10.1016/j.smim.2006.07.008 17011789

[45] Li SL Duo LN Wang HJ Dai W Zhou EH Xu YN Identification of LCK mutation in a family with atypical epidermodysplasia verruciformis with T-cell defects and virus-induced squamous cell carcinoma. Br J Dermatol (2016) 175(6):1204–9. 10.1111/bjd.14679 27087313

[46] Sorte HS Osnes LT Fevang B Aukrust P Erichsen HC Backe PH A potential founder variant in CARMIL2/RLTPR in three Norwegian families with warts, molluscum contagiosum, and T-cell dysfunction. Mol Genet Genomic Med (2016) 4(6):604–16. 10.1002/mgg3.237 27896283 PMC5118205

[47] Crequer A Troeger A Patin E Ma CS Picard C Pedergnana V Human RHOH deficiency causes T cell defects and susceptibility to EV-HPV infections. J Clin Invest (2012) 122(9):3239–47. 10.1172/JCI62949 22850876 PMC3428089

[48] Huppmann AR Leiding JW Hsu AP Raffeld M Uzel G Pittaluga S Pathologic findings in NEMO deficiency: a surgical and autopsy survey. Pediatr Dev Pathol (2015) 18(5):387–400. 10.2350/15-05-1631-OA.1 26230867

[49] Spinner MA Sanchez LA Hsu AP Shaw PA Zerbe CS Calvo KR GATA2 deficiency: a protean disorder of hematopoiesis, lymphatics, and immunity. Blood (2014) 123(6):809–21. 10.1182/blood-2013-07-515528 24227816 PMC3916876

[50] Hsu AP Johnson KD Falcone EL Sanalkumar R Sanchez L Hickstein DD GATA2 haploinsufficiency caused by mutations in a conserved intronic element leads to MonoMAC syndrome. Blood (2013) 121(19):3830–7, S1–7. 10.1182/blood-2012-08-452763 23502222 PMC3650705

[51] Hsu AP McReynolds LJ Holland SM. GATA2 deficiency. Curr Opin Allergy Clin Immunol (2015) 15(1):104–9. 10.1097/ACI.0000000000000126 25397911 PMC4342850

[52] Toubiana J Okada S Hiller J Oleastro M Lagos Gomez M Aldave Becerra JC Heterozygous STAT1 gain-of-function mutations underlie an unexpectedly broad clinical phenotype. Blood (2016) 127(25):3154–64. 10.1182/blood-2015-11-679902 27114460 PMC4920021

[53] Furio L Pampalakis G Michael IP Nagy A Sotiropoulou G Hovnanian A. KLK5 inactivation reverses cutaneous hallmarks of Netherton syndrome. PLoS Genet (2015) 11(9):e1005389. 10.1371/journal.pgen.1005389 26390218 PMC4577096

[54] Weber F Fuchs PG Pfister HJ Hintner H Fritsch P Hoepfl R. Human papillomavirus infection in Netherton’s syndrome. Br J Dermatol (2001) 144(5):1044–9. 10.1046/j.1365-2133.2001.04196.x 11359395

[55] Hannula-Jouppi K Laasanen SL Ilander M Furio L Tuomiranta M Marttila R Intrafamily and interfamilial phenotype variation and immature immunity in patients with Netherton syndrome and Finnish SPINK5 Founder Mutation. JAMA Dermatol (2016) 152(4):435–42. 10.1001/jamadermatol.2015.5827 26865388

[56] Abdollahpour H Appaswamy G Kotlarz D Diestelhorst J Beier R Schaffer AA The phenotype of human STK4 deficiency. Blood (2012) 119(15):3450–7. 10.1182/blood-2011-09-378158 22294732 PMC3325036

[57] Balabanian K Lagane B Pablos JL Laurent L Planchenault T Verola O WHIM syndromes with different genetic anomalies are accounted for by impaired CXCR4 desensitization to CXCL12. Blood (2005) 105(6):2449–57. 10.1182/blood-2004-06-2289 15536153

[58] Kawai T Malech HL. WHIM syndrome: congenital immune deficiency disease. Curr Opin Hematol (2009) 16(1):20–6. 10.1097/MOH.0b013e32831ac557 19057201 PMC2673024

[59] Beaussant Cohen S Fenneteau O Plouvier E Rohrlich PS Daltroff G Plantier I Description and outcome of a cohort of 8 patients with WHIM syndrome from the French Severe Chronic Neutropenia Registry. Orphanet J Rare Dis (2012) 7:71. 10.1186/1750-1172-7-71 23009155 PMC3585856

[60] Kreuter A Hochdorfer B Brockmeyer NH Altmeyer P Pfister H Wieland U A human papillomavirus-associated disease with disseminated warts, depressed cell-mediated immunity, primary lymphedema, and anogenital dysplasia: WILD syndrome. Arch Dermatol (2008) 144(3):366–72. 10.1001/archderm.144.3.366 18347293

[61] Kreuter A Waterboer T Wieland U Regression of cutaneous warts in a patient with WILD syndrome following recombinant quadrivalent human papillomavirus vaccination. Arch Dermatol (2010) 146(10):1196–7. 10.1001/archdermatol.2010.290 20956677

[62] Carlson JA Rohwedder A Daulat S Schwartz J Schaller J. Detection of human papillomavirus type 10 DNA in eccrine syringofibroadenomatosis occurring in Clouston’s syndrome. J Am Acad Dermatol (1999) 40(2 Pt 1):259–62. 10.1016/S0190-9622(99)70201-X 10025758

[63] Tobin E Rohwedder A Holland SM Philips B Carlson JA. Recurrent ‘sterile’ verrucous cyst abscesses and epidermodysplasia verruciformis-like eruption associated with idiopathic CD4 lymphopenia. Br J Dermatol (2003) 149(3):627–33. 10.1046/j.1365-2133.2003.05543.x 14511000

[64] Laffort C Le Deist F Favre M Caillat-Zucman S Radford-Weiss I Debre M Severe cutaneous papillomavirus disease after haemopoietic stem-cell transplantation in patients with severe combined immune deficiency caused by common gammac cytokine receptor subunit or JAK-3 deficiency. Lancet (2004) 363(9426):2051–4. 10.1016/S0140-6736(04)16457-X 15207958

[65] Kamili QU Seeborg FO Saxena K Nicholas SK Banerjee PP Angelo LS Severe cutaneous human papillomavirus infection associated with natural killer cell deficiency following stem cell transplantation for severe combined immunodeficiency. J Allergy Clin Immunol (2014) 134(6):1451–3.e1. 10.1016/j.jaci.2014.07.009 25159470 PMC5182041

[66] Sterling JC Gibbs S Haque Hussain SS Mohd Mustapa MF Handfield-Jones SE British Association of Dermatologists’ guidelines for the management of cutaneous warts 2014. Br J Dermatol (2014) 171(4):696–712. 10.1111/bjd.13310 25273231

[67] Karnes JB Usatine RP. Management of external genital warts. Am Fam Physician (2014) 90(5):312–8. 25251091

[68] Yanofsky VR Patel RV Goldenberg G. Genital warts: a comprehensive review. J Clin Aesthet Dermatol (2012) 5(6):25–36. 22768354 PMC3390234

[69] Lopaschuk CC. New approach to managing genital warts. Can Fam Physician (2013) 59(7):731–6. 23851535 PMC3710035

[70] Ozarmagan G Didem Yazganoglu K Agacfidan A. Hyper-IgE syndrome with widespread premalign oral papillomas treated with interferon alpha2b. Acta Derm Venereol (2005) 85(5):433–5. 10.1080/00015550510030041 16159737

[71] Gaspari AA Zalka AD Payne D Menegus M Bunce LA Abboud CN Successful treatment of a generalized human papillomavirus infection with granulocyte-macrophage colony-stimulating factor and interferon gamma immunotherapy in a patient with a primary immunodeficiency and cyclic neutropenia. Arch Dermatol (1997) 133(4):491–6. 10.1001/archderm.1997.03890400091012 9126013

[72] Henrickson SE Treat JR. Topical cidofovir for recalcitrant verrucae in individuals with severe combined immunodeficiency after hematopoietic stem cell transplantation. Pediatr Dermatol (2017) 34(1):e24–5. 10.1111/pde.12992 27699886 PMC5488717

[73] Wade NA Lepow ML Veazey J Meuwissen HJ. Progressive varicella in three patients with Wiskott-Aldrich syndrome: treatment with adenine arabinoside. Pediatrics (1985) 75(4):672–5. 3982899

[74] Siegel AM Heimall J Freeman AF Hsu AP Brittain E Brenchley JM A critical role for STAT3 transcription factor signaling in the development and maintenance of human T cell memory. Immunity (2011) 35(5):806–18. 10.1016/j.immuni.2011.09.016 22118528 PMC3228524

[75] Dorman SE Uzel G Roesler J Bradley JS Bastian J Billman G Viral infections in interferon-gamma receptor deficiency. J Pediatr (1999) 135(5):640–3. 10.1016/S0022-3476(99)70064-8 10547254 PMC7095028

[76] Azuma H Sakata H Saijyou M Okuno A. Effect of interleukin 2 on intractable herpes virus infection and chronic eczematoid dermatitis in a patient with Wiskott-Aldrich syndrome. Eur J Pediatr (1993) 152(12):998–1000. 10.1007/BF01957224 8131820

[77] Orange JS Roy-Ghanta S Mace EM Maru S Rak GD Sanborn KB IL-2 induces a WAVE2-dependent pathway for actin reorganization that enables WASp-independent human NK cell function. J Clin Invest (2011) 121(4):1535–48. 10.1172/JCI44862 21383498 PMC3069781

[78] Smith PM Wolcott RM Chervenak R Jennings SR. Control of acute cutaneous herpes simplex virus infection: T cell-mediated viral clearance is dependent upon interferon-gamma (IFN-gamma). Virology (1994) 202(1):76–88. 10.1006/viro.1994.1324 7912023

[79] Weiner N Williams N Birch G Ramachandran C Shipman C Jr Flynn G. Topical delivery of liposomally encapsulated interferon evaluated in a cutaneous herpes guinea pig model. Antimicrob Agents Chemother (1989) 33(8):1217–21. 10.1128/AAC.33.8.1217 2802550 PMC172628

[80] Shalev Y Berrebi A Green L Levin S Frumkin A Hurwitz N Progressive cutaneous herpes simplex infection in acute myeloblastic leukemia. Successful treatment with interferon and cytarabine. Arch Dermatol (1984) 120(7):922–6. 10.1001/archderm.1984.01650430108020 6587832

[81] Cardamakis E Relakis K Kotoulas IG Michopoulos J Metallinos K Mantouvalos H Treatment of recurrent genital herpes with interferon alpha-2alpha. Gynecol Obstet Invest (1998) 46(1):54–7. 10.1159/000009998 9692344

[82] Shupack J Stiller M Davis I Kenny C Jondreau L. Topical alpha-interferon ointment with dimethyl sulfoxide in the treatment of recurrent genital herpes simplex. Dermatology (1992) 184(1):40–4. 10.1159/000247497 1558994

[83] Shupack J Stiller M Knobler E Ackerman C Jondreau L Kenny C. Topical alpha-interferon in recurrent genital herpes simplex infection. A double-blind, placebo-controlled clinical trial. Dermatologica (1990) 181(2):134–8. 10.1159/000247902 2242782

[84] Laxmisha C Thappa DM Jaisankar TJ. Clinical profile of molluscum contagiosum in children versus adults. Dermatol Online J (2003) 9(5):1. 14996374

[85] Kilic SS Puel A Casanova JL. Orf infection in a patient with Stat1 gain-of-function. J Clin Immunol (2015) 35(1):80–3. 10.1007/s10875-014-0111-7 25367169

[86] National guideline for the management of molluscum contagiosum. Clinical effectiveness group (Association of Genitourinary Medicine and the Medical Society for the Study of Venereal Diseases). Sex Transm Infect (1999) 75(Suppl 1):S80–1. 10616392

[87] Nguyen HP Franz E Stiegel KR Hsu S Tyring SK. Treatment of molluscum contagiosum in adult, pediatric, and immunodeficient populations. J Cutan Med Surg (2014) 18(5):299–306. 10.2310/7750.2013.13133 25186990

[88] Bohm M Luger TA Bonsmann G. Disseminated giant molluscum contagiosum in a patient with idiopathic CD4+ lymphocytopenia. Successful eradication with systemic interferon. Dermatology (2008) 217(3):196–8. 10.1159/000141649 18583911

[89] Kilic SS Kilicbay F. Interferon-alpha treatment of molluscum contagiosum in a patient with hyperimmunoglobulin E syndrome. Pediatrics (2006) 117(6):e1253–5. 10.1542/peds.2005-2706 16651279

[90] Na CH Kim DJ Kim MS Kim JK Shin BS. Successful treatment of molluscum contagiosum with intralesional immunotherapy by measles, mumps, and rubella vaccine: a report of two cases. Dermatol Ther (2014) 27(6):373–6. 10.1111/dth.12158 25053017

[91] Perelygina L Plotkin S Russo P Hautala T Bonilla F Ochs HD Rubella persistence in epidermal keratinocytes and granuloma M2 macrophages in patients with primary immunodeficiencies. J Allergy Clin Immunol (2016) 138(5):1436–9.e11. 10.1016/j.jaci.2016.06.030 27613149 PMC5392721

[92] Bodemer C Sauvage V Mahlaoui N Cheval J Couderc T Leclerc-Mercier S Live rubella virus vaccine long-term persistence as an antigenic trigger of cutaneous granulomas in patients with primary immunodeficiency. Clin Microbiol Infect (2014) 20(10):O656–63. 10.1111/1469-0691.12573 24476349

[93] Neven B Perot P Bruneau J Pasquet M Ramirez M Diana JS Cutaneous and visceral chronic granulomatous disease triggered by a rubella virus vaccine strain in children with primary immunodeficiencies. Clin Infect Dis (2017) 64(1):83–6. 10.1093/cid/ciw675 27810866

[94] Kelsen JR Dawany N Moran CJ Petersen BS Sarmady M Sasson A Exome sequencing analysis reveals variants in primary immunodeficiency genes in patients with very early onset inflammatory bowel disease. Gastroenterology (2015) 149(6):1415–24. 10.1053/j.gastro.2015.07.006 26193622 PMC4853027

[95] Barmettler S Otani IM Minhas J Abraham RS Chang Y Dorsey MJ Gastrointestinal manifestations in X-linked agammaglobulinemia. J Clin Immunol (2017) 37:287–94. 10.1007/s10875-017-0374-x 28236219 PMC5414010

[96] Uzzan M Ko HM Mehandru S Cunningham-Rundles C. Gastrointestinal disorders associated with common variable immune deficiency (CVID) and chronic granulomatous disease (CGD). Curr Gastroenterol Rep (2016) 18(4):17. 10.1007/s11894-016-0491-3 26951230 PMC4837890

[97] Donaldson EF Lindesmith LC Lobue AD Baric RS. Norovirus pathogenesis: mechanisms of persistence and immune evasion in human populations. Immunol Rev (2008) 225:190–211. 10.1111/j.1600-065X.2008.00680.x 18837783

[98] Desselberger U Huppertz HI Immune responses to rotavirus infection and vaccination and associated correlates of protection. J Infect Dis (2011) 203(2):188–95. 10.1093/infdis/jiq031 21288818 PMC3071058

[99] Green KY. Norovirus infection in immunocompromised hosts. Clin Microbiol Infect (2014) 20(8):717–23. 10.1111/1469-0691.12761 25040790 PMC11036326

[100] Frange P Touzot F Debre M Heritier S Leruez-Ville M Cros G Prevalence and clinical impact of norovirus fecal shedding in children with inherited immune deficiencies. J Infect Dis (2012) 206(8):1269–74. 10.1093/infdis/jis498 22872736

[101] Henke-Gendo C Harste G Juergens-Saathoff B Mattner F Deppe H Heim A. New real-time PCR detects prolonged norovirus excretion in highly immunosuppressed patients and children. J Clin Microbiol (2009) 47(9):2855–62. 10.1128/JCM.00448-09 19625473 PMC2738087

[102] Woodward JM Gkrania-Klotsas E Cordero-Ng AY Aravinthan A Bandoh BN Liu H The role of chronic norovirus infection in the enteropathy associated with common variable immunodeficiency. Am J Gastroenterol (2015) 110(2):320–7. 10.1038/ajg.2014.432 25623655

[103] Razvi S Schneider L Jonas MM Cunningham-Rundles C. Outcome of intravenous immunoglobulin-transmitted hepatitis C virus infection in primary immunodeficiency. Clin Immunol (2001) 101(3):284–8. 10.1006/clim.2001.5132 11726220

[104] Centers for Disease Control and Prevention. Outbreak of hepatitis C associated with intravenous immunoglobulin administration – United States, October 1993–June 1994. MMWR Morb Mortal Wkly Rep (1994) 43(28):505–9. 8022396

[105] Bjoro K Froland SS Yun Z Samdal HH Haaland T. Hepatitis C infection in patients with primary hypogammaglobulinemia after treatment with contaminated immune globulin. N Engl J Med (1994) 331(24):1607–11. 10.1056/NEJM199412153312402 7526215

[106] Chapel HM Christie JM Peach V Chapman RW. Five-year follow-up of patients with primary antibody deficiencies following an outbreak of acute hepatitis C. Clin Immunol (2001) 99(3):320–4. 10.1006/clim.2001.5036 11358426

[107] Stiehm ER. Adverse effects of human immunoglobulin therapy. Transfus Med Rev (2013) 27(3):171–8. 10.1016/j.tmrv.2013.05.004 23835249

[108] Saulsbury FT Winkelstein JA Yolken RH. Chronic rotavirus infection in immunodeficiency. J Pediatr (1980) 97(1):61–5. 10.1016/S0022-3476(80)80131-4 6247473

[109] Gilger MA Matson DO Conner ME Rosenblatt HM Finegold MJ Estes MK. Extraintestinal rotavirus infections in children with immunodeficiency. J Pediatr (1992) 120(6):912–7. 10.1016/S0022-3476(05)81959-6 1317419

[110] Bakare N Menschik D Tiernan R Hua W Martin D. Severe combined immunodeficiency (SCID) and rotavirus vaccination: reports to the vaccine adverse events reporting system (VAERS). Vaccine (2010) 28(40):6609–12. 10.1016/j.vaccine.2010.07.039 20674876

[111] Patel NC Hertel PM Estes MK de la Morena M Petru AM Noroski LM Vaccine-acquired rotavirus in infants with severe combined immunodeficiency. N Engl J Med (2010) 362(4):314–9. 10.1056/NEJMoa0904485 20107217 PMC4103739

[112] Fiore L Plebani A Buttinelli G Fiore S Donati V Marturano J Search for poliovirus long-term excretors among patients affected by agammaglobulinemia. Clin Immunol (2004) 111(1):98–102. 10.1016/j.clim.2003.11.011 15093557

[113] Galal NM Bassiouny L Nasr E Abdelmeguid N. Isolation of poliovirus shedding following vaccination in children with antibody deficiency disorders. J Infect Dev Ctries (2012) 6(12):881–5. 10.3855/jidc.2372 23276742

[114] Halsey NA Pinto J Espinosa-Rosales F Faure-Fontenla MA da Silva E Khan AJ Search for poliovirus carriers among people with primary immune deficiency diseases in the United States, Mexico, Brazil, and the United Kingdom. Bull World Health Organ (2004) 82(1):3–8. 15106294 PMC2585894

[115] Melamed I Griffiths AM Roifman CM Benefit of oral immune globulin therapy in patients with immunodeficiency and chronic diarrhea. J Pediatr (1991) 119(3):486–9. 10.1016/S0022-3476(05)82070-0 1880668

[116] Losonsky GA Johnson JP Winkelstein JA Yolken RH. Oral administration of human serum immunoglobulin in immunodeficient patients with viral gastroenteritis. A pharmacokinetic and functional analysis. J Clin Invest (1985) 76(6):2362–7. 10.1172/JCI112248 4077983 PMC424377

[117] McGavern DB Kang SS Illuminating viral infections in the nervous system. Nat Rev Immunol (2011) 11(5):318–29. 10.1038/nri2971 21508982 PMC5001841

[118] Rantakallio P Leskinen M von Wendt L. Incidence and prognosis of central nervous system infections in a birth cohort of 12,000 children. Scand J Infect Dis (1986) 18(4):287–94. 10.3109/00365548609032339 3764348

[119] Kelly TA O’Lorcain P Moran J Garvey P McKeown P Connell J Underreporting of viral encephalitis and viral meningitis, Ireland, 2005–2008. Emerg Infect Dis (2013) 19(9):1428–36. 10.3201/eid1909.130201 23965781 PMC3810922

[120] Raschilas F Wolff M Delatour F Chaffaut C De Broucker T Chevret S Outcome of and prognostic factors for herpes simplex encephalitis in adult patients: results of a multicenter study. Clin Infect Dis (2002) 35(3):254–60. 10.1086/341405 12115090

[121] Zhang SY Abel L Casanova JL. Mendelian predisposition to herpes simplex encephalitis. Handb Clin Neurol (2013) 112:1091–7. 10.1016/B978-0-444-52910-7.00027-1 23622315

[122] Lim HK Seppanen M Hautala T Ciancanelli MJ Itan Y Lafaille FG TLR3 deficiency in herpes simplex encephalitis: high allelic heterogeneity and recurrence risk. Neurology (2014) 83(21):1888–97. 10.1212/WNL.0000000000000999 25339207 PMC4248460

[123] Abel L Plancoulaine S Jouanguy E Zhang SY Mahfoufi N Nicolas N Age-dependent Mendelian predisposition to herpes simplex virus type 1 encephalitis in childhood. J Pediatr (2010) 157(4):623–9, 629.e1. 10.1016/j.jpeds.2010.04.020 20553844

[124] Bearden D Collett M Quan PL Costa-Carvalho BT Sullivan KE. Enteroviruses in X-linked agammaglobulinemia: update on epidemiology and therapy. J Allergy Clin Immunol Pract (2016) 4(6):1059–65. 10.1016/j.jaip.2015.12.015 26883540

[125] Minor PD. Comparative biochemical studies of type 3 poliovirus. J Virol (1980) 34(1):73–84. 6246264 10.1128/jvi.34.1.73-84.1980 PMC288672

[126] Nkowane BM Wassilak SG Orenstein WA Bart KJ Schonberger LB Hinman AR Vaccine-associated paralytic poliomyelitis. United States: 1973 through 1984. JAMA (1987) 257(10):1335–40. 10.1001/jama.257.10.1335 3029445

[127] MacCallum FO Hypogammaglobulinaemia in the United Kingdom. VII. The role of humoral antibodies in protection against and recovery from bacterial and virus infections in hypogammaglobulinaemia. Spec Rep Ser Med Res Counc (G B) (1971) 310:72–85. 4324778

[128] Etsano A Damisa E Shuaib F Nganda GW Enemaku O Usman S Environmental isolation of circulating vaccine-derived poliovirus after interruption of wild poliovirus transmission – Nigeria, 2016. MMWR Morb Mortal Wkly Rep (2016) 65(30):770–3. 10.15585/mmwr.mm6530a4 27490081

[129] Kew O Morris-Glasgow V Landaverde M Burns C Shaw J Garib Z Outbreak of poliomyelitis in Hispaniola associated with circulating type 1 vaccine-derived poliovirus. Science (2002) 296(5566):356–9. 10.1126/science.1068284 11896235

[130] Jorba J Diop OM Iber J Sutter RW Wassilak SG Burns CC Update on vaccine-derived polioviruses – worldwide, January 2015–May 2016. MMWR Morb Mortal Wkly Rep (2016) 65(30):763–9. 10.15585/mmwr.mm6530a3 27491079

[131] Guo J Bolivar-Wagers S Srinivas N Holubar M Maldonado Y. Immunodeficiency-related vaccine-derived poliovirus (iVDPV) cases: a systematic review and implications for polio eradication. Vaccine (2015) 33(10):1235–42. 10.1016/j.vaccine.2015.01.018 25600519 PMC5529169

[132] Wintergerst U Belohradsky BH. Acyclovir monotherapy versus acyclovir plus beta-interferon in focal viral encephalitis in children. Infection (1992) 20(4):207–12. 10.1007/BF02033060 1521886

